# Effect of passive ultrasonic activation on microorganisms in primary root canal infection: a randomized clinical trial

**DOI:** 10.1590/1678-7757-2019-0100

**Published:** 2019-11-15

**Authors:** Esteban Isai Flores Orozco, Cassia Cestari Toia, Daiana Cavalli, Rayana Duarte Khoury, Flávia Goulart da Rosa Cardoso, Eduardo Bresciani, Marcia Carneiro Valera

**Affiliations:** 1 Universidade Estadual Paulista (UNESP), Instituto de Ciências e Tecnologia, Departamento de Odontologia Restauradora, São José dos Campos, São Paulo, Brasil

**Keywords:** Root canal therapy, Bacteria, Ultrasound

## Abstract

**Objective:**

This clinical study sought to evaluate the effectiveness of passive ultrasonic activation (PUA) in eliminating microorganisms in primary endodontic infection (PEI) after instrumentation of root canals using microbiological culture and checkerboard DNA-DNA hybridization.

**Methodology:**

Twenty root canals with PEI and apical periodontitis were selected. The root canals were instrumented and then randomly divided into 2 groups, according to the irrigation method: PUA and conventional needle irrigation (CNI). Microbiological samples were collected before instrumentation (S1), after instrumentation (S2) and after irrigation with 17% EDTA (S3). The samples were subjected to anaerobic culture technique and checkerboard DNA-DNA hybridization analysis.

**Results:**

A statistically significant difference was found between CNI (23.56%) and PUA (98.37%) regarding the median percentage values for culturable bacteria reduction (p<0.05). In the initial samples, the most frequently detected species was *S. constellatus* (50%), and after root canal treatment was *E. faecalis* (50%).

**Conclusion:**

Both treatments significantly decreased the number of bacterial species compared with the initial sample. However, no statistical difference in the total microbial load between PUA and CNI groups was detected. The number of cultivable anaerobic bacteria reduced significantly using PUA, and the bacterial composition and number of bacterial species after using either CNI or PUA was similar.

## Introduction

The successful treatment of apical periodontitis depends on the maximum decrease in microorganisms and their by-products in root canals. Root canal preparation is associated with an irrigating solution to obtain maximal reduction in microbial load inside the root canal to prevent or eliminate apical periodontitis.^1^^–^^3^ The irrigant used during instrumentation is supposed to act as a cleaning, disinfectant and lubricant agent.^4^ Sodium hypochlorite (NaOCl) is the most widely used irrigant in endodontics, especially due to its antimicrobial activity^6^ and organic tissue dissolution capacity.^4^^,^^5^ In addition to NaOCl, the use of ethylenediaminetetraacetic acid (EDTA) is a common practice in endodontic treatment to remove the inorganic component or smear layer left in the canal during endodontic treatment.^6^

However, the root canal system has some anatomical complexities such as apical ramifications, isthmus, and dentinal tubules, which may impede full disinfection. Studies have shown the presence of microorganisms in necrotic teeth not only in the main canal, but also throughout the root canal system, even after chemomechanical preparation.^7^^–^^10^ The remaining bacteria may influence the treatment result and can be associated with persistent apical periodontitis.^11^ Thus, all efforts have been made to obtain maximum bacterial elimination from the root canals before filling.^3^

Conventional needle irrigation (CNI) is the most commonly performed irrigation system worldwide. Despite its good control over the irrigant delivery, this technique seems to be unable to flush out organic and inorganic tissue remnants and to clean the apical third of the root canal.^12^ Several adjunctive approaches have been developed to overcome the limitations of CNI. Passive ultrasonic activation (PUA) has been suggested to enhance root canal disinfection.^4^ This technique improves the cleanliness of instrumented and uninstrumented areas using ultrasonic activation of the irrigant, which is expected to aid the delivery of irrigants into difficult-to-reach areas.^13^

Despite the existence of several *ex vivo* studies assessing the antimicrobial effect of ultrasonic activation with NaOCl as an adjunctive step, they have been inconclusive regarding bacterial load reduction. While some studies demonstrate better efficacy using the ultrasonic activation protocol,^14^ others report absence of significant difference.^15^^,^^16^ In clinical study, randomized clinical trials are the best way to study the safety and efficacy of new interventions and treatments.^17^ Only a few clinical studies have evaluated the effectiveness of the ultrasonic activation approach in improving significantly the disinfection after biomechanical procedure.^18^^,^^19^ However, the extrapolation of outcomes from *ex vivo* studies to the clinical settings must be prudent. Although the complete eradication of microorganisms does not occur, some studies have reported a high frequency of negative cultures,^19^ which may be related to the limitations of culture methods, including low sensitivity and inability to detect non-cultivable bacterial species.^20^ Therefore, using molecular approaches is essential to analyze antimicrobial effects of endodontic procedures to overcome such issues, also providing a more accurate sight of the microbiological conditions.^20^^,^^21^

Only one randomized clinical trial assessing the antimicrobial effect of ultrasonic activation by molecular-based methods has been reported.^18^ Therefore, this randomized clinical study sought to assess the antibacterial effects of final irrigation protocols using PUA or CNI after biomechanical preparation with single-file reciprocation technique, using 2.5% NaOCl, in teeth with primary endodontic infection using culture and molecular-based methods. Considering the advantage of volumetric analysis of bone destruction determined by Cone Beam Computed Tomography (CBCT) image, this clinical study also assessed the clinical outcomes by measuring the periapical lesion reduction with a 18-month follow-up by CBCT analysis.

## Methodology

### Patient selection

**Figure 1:**
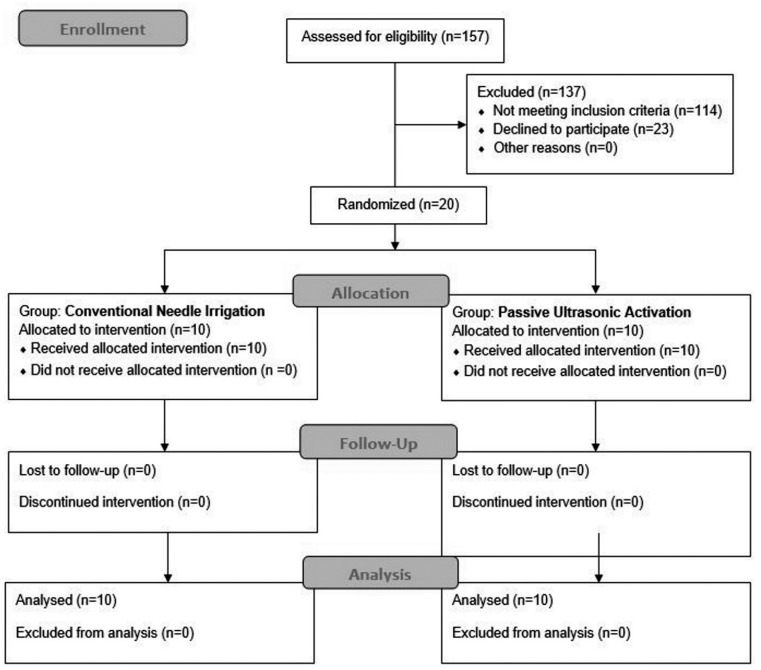
CONSORT flow diagram

This randomized clinical trial was performed at São José dos Campos Dental School, São José dos Campos, SP, Brazil, and was approved by the local Institutional Review Board (CEP: 83576418.0.0000.0077). The clinical trial was registered at the Brazilian Clinical Trials Registry (http://ensaiosclinicos.gov.br) Primary ID:RBR-7CXWG5. Considering a 99% reduction in the anaerobic bacteria count^22^ as a standard reduction; 5% significance level, 80% power, equivalence limit of 15% and sample size of 10 patients *per* group were required. Power was estimated using the website https://sealedenvelope.com/power/binary-equivalence/, under binary outcome and equivalence trial option. From 157 patients examined, 20 requiring primary endodontic treatment were selected for this study (Figure 1). The study included upper and lower single rooted teeth having primary endodontic infection with radiographic evidence of apical periodontitis and intact pulp chamber walls. The diagnosis of pulpal necrosis was confirmed by a negative response to the cold test. Patient age ranged from 18 to 60 years (50% were male and 50% female). Among the 20 unirradicular teeth included in the study, 7 were upper lateral incisors (7/20), 3 lower central incisors (3/20), 3 lower lateral incisor (3/20), 2 lower canines (2/20), 3 lower first premolars (3/20) and 2 lower second premolars (2/20). All patients were volunteers and signed an informed consent form. The exclusion criteria were: tooth that could not be isolated with rubber dam, tooth with periodontal pockets deeper than 4 mm, and patients who had received antibiotic treatment during the past 3 months or had any general disease. A detailed dental history was requested from each patient.

Clinical signs and symptoms were recorded, and all patients included were subjected to Cone Beam Computed Tomography (CBCT) to determine the volume, in cubic millimeters, of periapical bone resorption before the endodontic treatment. Outcome measures were defined by periapical lesion volume reduction after a 18-month follow-up, and sign and symptom remission (Figure 6).

### Sampling procedures

Instruments and all materials used in this study were treated with ^60^Co gamma radiation (20 kGy for 6 hours) (EMBRARAD, Cotia, SP, Brazil). Samples were collected under aseptic conditions. The tooth was isolated with a rubber dam and had its crown and surrounding structures disinfected with 30% H_2_O_2_ [volume/volume (V/V)] for 30 seconds, followed by 5.25% NaOCl for the same period and then inactivated with 5% sodium thiosulfate.^9^

A two-stage access preparation was performed without using water spray but under manual irrigation with sterile/apyrogenic saline solution by using a sterile/apyrogenic high-speed diamond bur. The first stage was performed to promote a major removal of contaminants, including carious lesion and restoration. In the second stage, the access cavity was again disinfected with 5.25% NaOCl and subsequently inactivated with 5% sodium thiosulfate before entering the pulp chamber. All procedures were performed aseptically.

Root canal samples were taken as follows: 3 sterile paper points were consecutively introduced into the full length of the canal, which was determined radiographically, and retained in position for 60 seconds, and then immediately placed into a sterile tube containing 1 mL VMGA III (Viability Medium Göteborg Agar) transport medium for microbiologic analysis.^23^

After the first sample (S1 – baseline), the instrumentation was performed by one single operator using single-file reciprocation technique. According to the manufacturer's instructions, Reciproc R40 files (40.06) (VDW GmbH, Munich, Germany) were selected after confirming that for all teeth included the initial apical instrument was ISO size #20 hand file, which reached passively to working length. The file was adapted to an electric motor (VDW Silver, VDW GmbH, Munich, Germany) using preset adjustments. The instrument was introduced into the root canal until resistance was felt and then activated. Next, the instrument was apically moved using in-and-out pecking motions, with approximately 3 mm in amplitude by using light apical pressure. After 3 pecking motions, the instrument was removed and cleaned. Between each third (cervical, middle, and apical), 8 mL of 2.5% NaOCl was used to neutralize the content inside the root canal. The working length (WL) (-1 mm) was determined by using an apex locator (RomiApex A-15; Romidan Dental Solution, Kiryat-Ono, Israel) and confirmed by a periapical digital radiograph. Likewise, apical debridement was performed with a K-file size #30, which was extended 1 mm beyond this area. The root canal instrumentation was completed in a single visit in all cases, with a total of 24 mL of 2.5% NaOCl in both groups.

Subsequently, the patients were randomly distributed before receiving endodontic treatment with either CNI or PUA technique. The participants were divided into 2 groups by using the simple randomization method according to the CONSORT 2010 (Consolidated Standards of Reporting Trials),: an independent researcher prepared envelopes, including writing the technique name (either CNI or PUA) on a sheet of paper inside. Another researcher opened the envelope just before the procedure and informed the operator to perform the treatment with the technique written on the paper. All participants and laboratory raters were kept blind.

#### PUA Group

**Figure 2:**
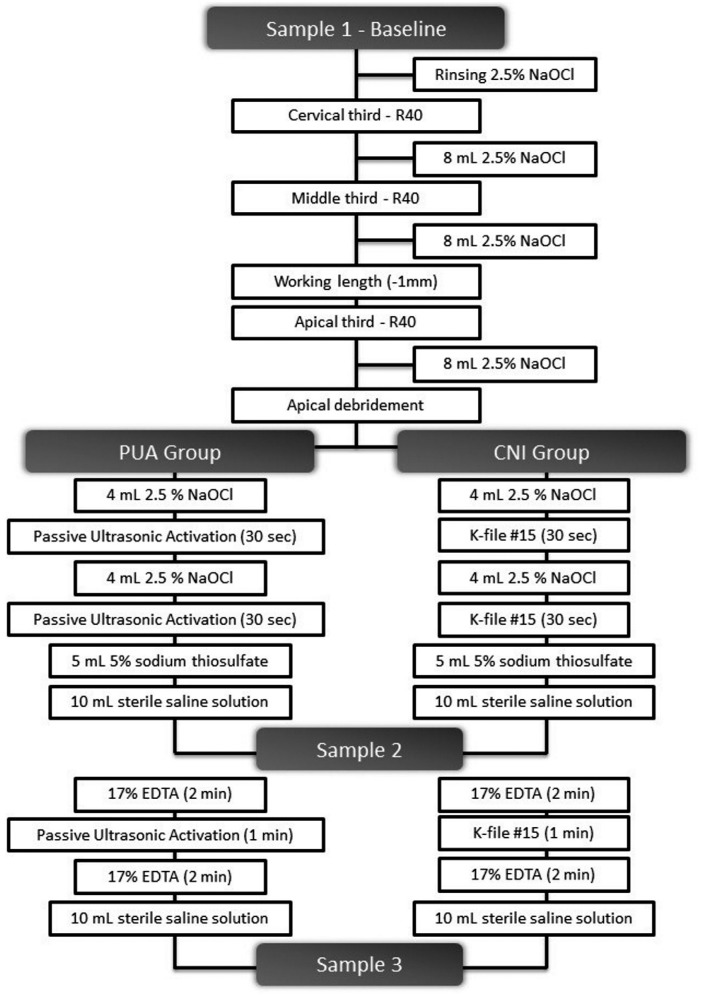
Flow diagram of interventions and sampling procedures

The root canals were irrigated with 4 mL of 2.5% NaOCl delivered by using a 31 gauge × 27 mm side port needle (NaviTip, Ultradent Products Inc., South Jordan, UT, USA) inserted up to 1 mm short of the WL, with PUA being performed for 30 seconds. The irrigating solution was renewed with 4 mL of 2.5% NaOCl and PUA was resumed for 30 additional seconds. For inactivation of 2.5% NaOCl, the canal was irrigated with 5 mL of 5% sodium thiosulfate, followed by irrigation with 10 mL of saline solution. The second sample was collected (S2) in the same manner as the first sample (S1). The smear layer was removed by rinsing the canal with 17% EDTA, which remained inside the canal for 2 minutes and then was activated with PUA for 1 minute. After additional 2 minutes inside the root canal, 17% EDTA was removed by irrigation with 10 mL of saline solution. After the procedure, the third sample was collected (S3). The ultrasonic activation was performed with a #20:01 non-cutting tip (E1 Irrisonic, Helse, São Paulo, SP, Brazil) and piezoelectric ultrasonic device (ALT – Equipamentos Médicos e Odontológicos, Ribeirão Preto, SP, Brazil) at 1000 Hz low power. The ultrasonic instrument was used at 1 mm short of the WL, avoiding contact with the root canal walls (Figure 2).

#### CNI Group

Similar to the PUA protocol; in this group, root canal was irrigated with 8 mL of 2.5% NaOCl by using a 31 gauge × 27 mm side port needle (NaviTip, Ultradent, South Jordan, UT, USA), inserted up to 1 mm short of the WL, and 17% EDTA remained inside the root canal for 4 minutes and manually agitated for 1 additional minute. The 2.5% NaOCl inactivation and 17% EDTA removal were performed in the same manner as described for PUA group. No ultrasonic activation was performed in this group (Figure 2).

Each group received a total of 8 mL of 2.5% NaOCl in the final irrigation protocol, and 17% EDTA remained inside the root canal for 5 minutes (Figure 2).

### Culture procedure (CFU/mL)

The transport media containing the root canal samples were thoroughly shaken for 60 seconds (Vortex; Marconi, Piracicaba, SP, Brazil). Serial 10-fold dilutions were made up to 10^−3^. Fifty microliters of the serial dilutions were plated onto 5% defibrinated sheep blood fastidious anaerobe agar (FAA; Lab M, Bury, UK) by using sterile plastic spreaders to culture nonselective obligate anaerobes and facultative anaerobes. The plates were incubated at 37°C in anaerobic atmosphere for up to 14 days. After this period, colony-forming units (CFUs) were visually quantified for each plate.

### Microbiological assessment: checkerboard DNA-DNA hybridization

Three hundred microliters of VMGA containing the root canal samples was transferred to another sterile tube. Subsequently, the tubes were centrifuged at 8000 rpm for 5 minutes. The supernatant was then discarded, and the pellet resuspended at 150 mL Tris-EDTA buffer [10 mmol/L tris (hydroxymethyl) aminomethane (Tris)-HCl, 1 mmol/L EDTA, pH=7.6]. Next, 100 mL 0.5 mol/L NaOH was added to each tube, and the samples were frozen at −20°C until they were processed.

**Figure 3:**
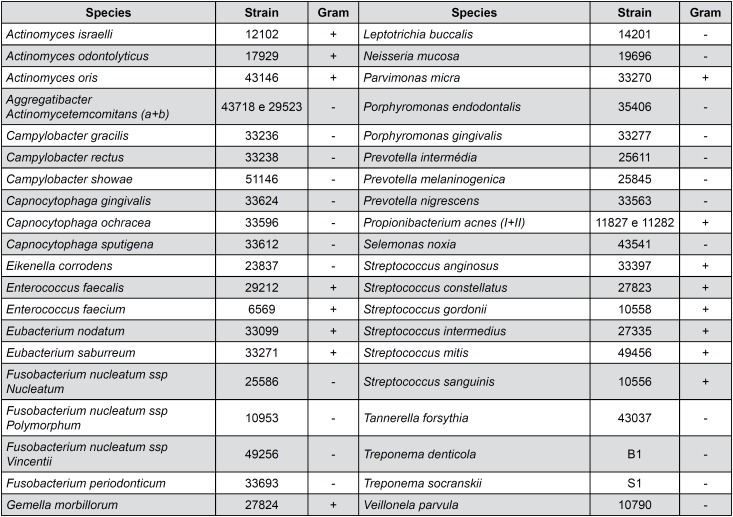
Bacterial strains used for the development of DNA probes

Presence, levels, and proportions of 40 bacterial species (Figure 3) were determined by the checkerboard DNA-DNA hybridization method described by Socransky, et al.^24^ (1994). The DNA probes were prepared by using the DIG DNA Labeling Kit (Roche Diagnostics, Indianapolis, IN, USA) and frozen until use. Next, the samples were boiled for 10 minutes, and 800 mL of 5 mol/L ammonium acetate were added to promote bacterial lyses and consequent suspension of DNA in solution. A nylon membrane (15×15 cm) with a positive charge (Amersham Biosciences, Chicago, IL, USA) was placed in a MiniSlot 30 apparatus (Immunetics, Cambridge, MA, USA), and 1000 mL of each suspension was placed into the extended slots of the MiniSlot 30 and fixed to the membrane by baking it at 120°C for 20 minutes. In each membrane, 28 samples were placed, and the last 2 channels of the MiniSlot 30 were reserved for placement of controls, containing a mixture of species of microorganisms that have been investigated by DNA probes at 2 concentrations (i.e., 10^5^ and 10^6^) of bacterial cells. A Miniblotter 45 apparatus (Immunetics, Cambridge, MA, USA) was used to hybridize the digoxigenin-labeled whole-genomic DNA probes perpendicular to the lanes of the clinical samples. Bound probes were detected using phosphatase conjugated antibodies to digoxigenin and chemiluminescence (CDP-Star Detection Reagent, GE Healthcare Limited, Chicago, IL, USA). The membranes were placed under a radiographic film (AGFA-IBF, Duque de Caxias, RJ, Brazil) for almost 60 minutes. The films were processed right after. Each probe produced a certain type of signal, which was visually compared with the signals produced by the probes in the 2 controls containing 10^5^ and 10^6^ bacterial cells. The signals were coded into 6 different classes according to the following count levels: 0: not detected, 1: <10^5^ cells, 2: nearly 10^5^ cells, 3: between 10^5^ and 10^6^ cells.

### CBCT analysis: periapical lesion volume (cubic millimeters)

The CBCT scannings were performed using the i-cat Classic (Imaging Science International, Hatfield, PA, USA) at São Paulo State University (UNESP), Institute of Science and Technology, São José dos Campos, Brazil. The volume of periapical bone resorption was quantified by following the reconstruction parameters previously described by Cardoso, et al.^8^ (2015).

### Statistical analysis

Data were typed into an Excel (Microsoft, Redmond, WA, USA) spreadsheet twice and analyzed with the STATISTICA 8.0 software (StatSoft Inc, Tulsa, OK, USA). Data did not present normal distribution, thus General Linear Model test was used to compare the effectiveness of endodontic treatment along the three sampling stages, in terms of the number of CFUs/mL. Regarding the number of detected bacterial species, data presented normal distribution and were subjected to 2-way repeated ANOVA test. The total bacterial load was analyzed by the General Linear Model. Multiple comparison tests were performed when detecting differences. For all tests, 5% significance level, p<0.05, was considered.

## Results

### Culture procedure

Bacteria were found in all initial samples (20/20), with median values of 2.4×10^5^ CFU/mL (20 – 1.8×10^6^ CFU/mL). A statistically significant difference was found in the median percentage values for the reduction in cultivable bacteria (p<0.05) between CNI (23.56%) and PUA (98.37%), producing 30% and 80% root canals free of cultivable bacteria in CNI and PUA group, respectively, in endodontic treatment (S3). No differences were found between S2 and S3 (p>0.05). The analysis results (CFUs/mL) are shown in Table 1.

**Table 1:** Effectiveness of PUA and CNI protocol in removing cultivable bacteria (CFUs/mL) in primary endodontic infection (Uppercase – Different columns; lowercase – different rows)

Final Irrigation Protocol	Cultivable Bacteria (CFUs/mL) – Mean ± SD		
	Before treatment (S1)	After NaOCl irrigation (S2)	After EDTA irrigation (S3)
PUA*	2.58×10^5^ ± 4.70×10^5Aa^	6 ± 19^Bb^	42 ± 119^Bb^
CNI**	2.31×10^5^ ± 4.70×10^5Aa^	5.72×10³ ± 1.10×10^4Ab^	1.76×10³ ± 3.31×10^3Ab^

* PUA - Passive Ultrasonic Activation;

** CNI - Conventional Needle Irrigation

### Checkerboard DNA-DNA hybridization

**Figure 4:**
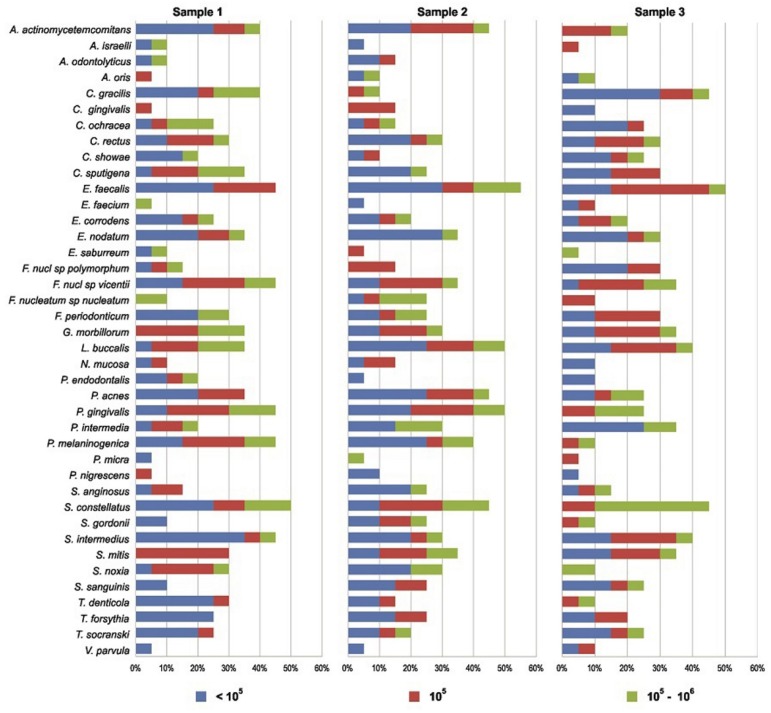
Stacked bar chart of frequency and DNA concentration of individual bacterial species investigated before root canal treatment (S1), and after instrumentation (S2 and S3). The total length of each bar indicates the percentage of positive samples. Different colors within each bar indicate the percentage of samples containing different concentrations of bacterial DNA

The results of the checkerboard DNA-DNA analysis revealed that the 40 DNA bacterial probes tested were reactive with at least 1 or more clinical samples in S1. All root canals investigated showed bacterial signals for at least 1 of the 40 DNA bacterial probes tested in S1 (baseline), with 1 to 18 (mean=9.6) bacterial species *per* root canal. The most frequently detected species were *S. constellatus* (50%), *E. faecalis* (45%), *F. nucleatum SP* (45%), *P. gingivalis* (45%), *P. melaninogenica* (45%) and *S. intermedius* (45%). Frequency and DNA concentration of the each bacterial species investigated in S1 are shown in Figure 4. The mean number of bacterial species in S1 was 9±3.8 and 10.2±5.9, respectively, when comparing PUA with CNI (Table 2). The number of bacterial species ranged from 1 to 23 (mean=9.6) in samples collected after biomechanical preparation with single-file reciprocation technique using 2.5% NaOCl (S2). The most frequently detected species was *E. faecalis* (55%), *L. buccalis* (50%), *P. gingivalis* (50%), *A. actinomycetemcomitans* (45%), *P. acnes* (45%) and *S. constellatus* (45%). No statistical difference was observed in the number of detected species or in the total bacterial load between S1 and S2 (p>0.05). Frequency and DNA concentration of each bacterial species investigated in S2 are shown in Figure 4. The mean number of bacterial species in S2 was 10.7±6.7 and 8.6±6.9, respectively, when comparing PUA and CNI, as shown in Table 2. *S. constellatus, P. gingivalis* and *A. actinomycetemcomitans* were the most frequently detected species in PUA group; and *F. nucleatum sp. vicentii, L. buccalis* and *S. mitis* in CNI group. No statistical difference was detected (p>0.05).

**Table 2:** Effectiveness of PUA and CNI protocol in removing bacterial species in primary endodontic infection (Uppercase – Different columns; lowercase – different rows)

Final Irrigation Protocol	Number of Bacterial Species – Mean ± SD		
	Checkerboard DNA-DNA Hybridization		
	Before treatment (S1)	After NaOCl irrigation (S2)	After EDTA irrigation (S3)
PUA*	9 ± 3.8^Aa^	10.7 ± 6.7^Aa^	7.6 ± 5.5^Aa^
CNI**	10.2 ± 5.9^Aa^	8.6 ± 6.9^Aa^	9.8 ± 6.3^Aa^

* PUA - Passive Ultrasonic Activation;

** CNI - Conventional Needle Irrigation

**Figure 5:**
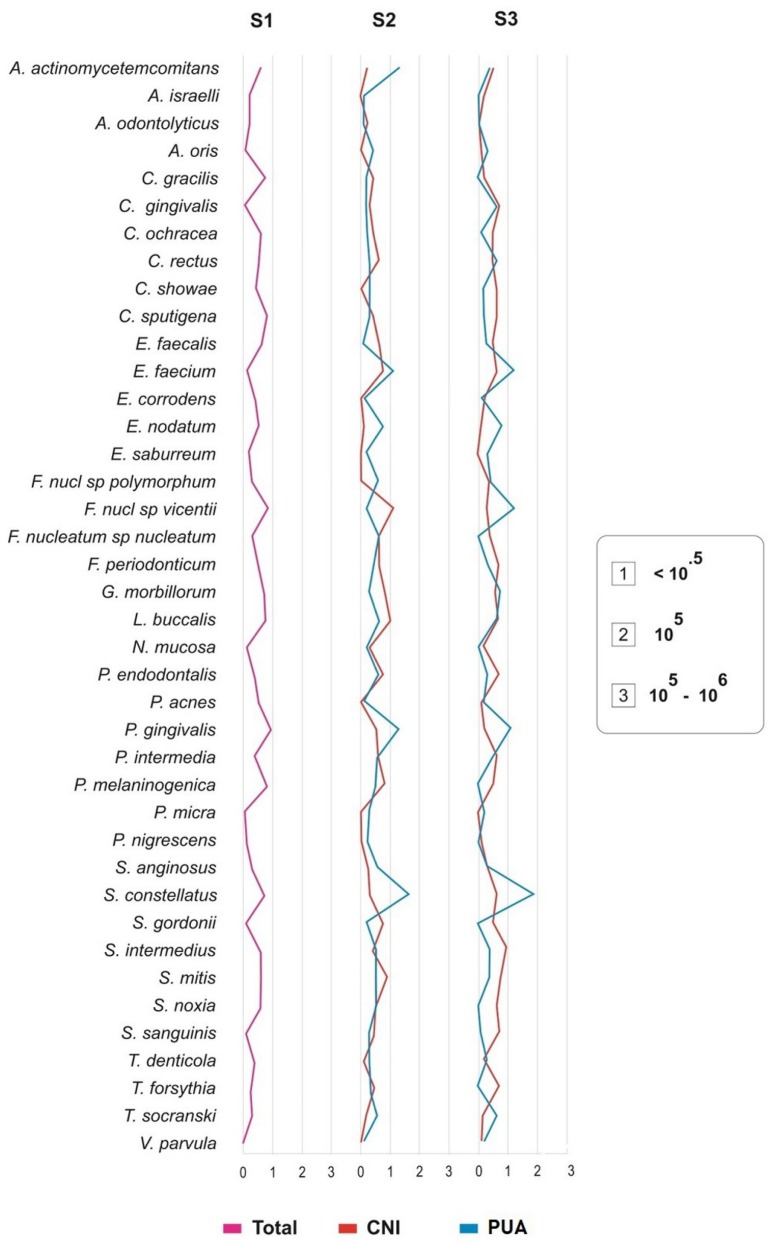
Bacterial levels before root canal treatment (S1), after instrumentation with PUA* or CNI** (S2) and after final irrigation using EDTA with PUA or CNI (S3)

After endodontic treatment, S3, the number of bacterial species ranged from 1 to 23 (mean=8.7). *E. faecalis was* the most frequently detected species *(50%).* No statistical difference in the number of detected species or the total bacterial load between S2 and S3 (p>0.05) was observed. The mean number of bacterial species in S3 was 7.6±5.5 and 9.8±6.3, respectively, when comparing PUA with CNI (Table 2), without statistical difference between the groups (p>0.05). Figure 5 shows the difference between the groups in the prevalence of microorganisms. A significantly greater reduction in the number of bacterial species and in the total bacterial load was observed in the final sample (S3) with the use of PUA protocol, completely eliminating 14 bacterial species when comparing with CNI group, which completely eliminated only 5 species.

**Figure 6:**
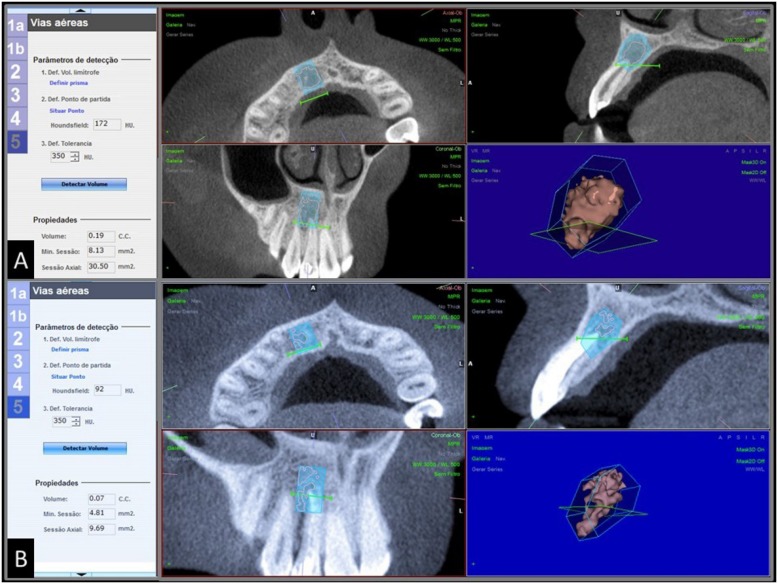
DICOM data of (A) preoperative and (B) 18-month follow-up analysis transferred to NEMOTEC^®^ software (Madrid, Spain), and 3D reconstruction of periapical lesion

### CBCT analysis: periapical lesion volume (cubic millimeters)

The measurement of the outcome was defined by CBCT analysis with a 18-month follow-up. Four patients from the PUA group were absent from the recall visits. The reduction was detected for both treatments (p<0.043), considering the initial lesion volume. The mean final volumes were 39.0±45.3 and 39.3±27.9, for CNI and PUA, respectively. No differences between the groups were detected at this follow-up (p=0.614) (Table 3).

**Table 3:** Effectiveness of PUA and CNI protocol in reducing periapical lesion volume in primary endodontic infection [Different letters mean statistically significant differences (p<0.05). Uppercase letters indicate difference in the line (intra-groups) and lowercase letters indicate difference in the column (inter-groups)]

Final Irrigation Protocol	CBCT Analysis	
	Initial Periapical Lesion Volume	18-month follow-up Periapical Lesion Volume
PUA*	63.3 ± 67.4^Aa^	39.3 ± 27.9^Ba^
CNI**	88.0 ± 72.2^Aa^	39.0 ± 45.3^Ba^

* PUA - Passive Ultrasonic Activation;

** CNI - Conventional Needle Irrigation

## Discussion

In this randomized clinical trial, PUA or CNI were assessed as final irrigation protocols. PUA has been suggested as an adjunctive procedure to increase the tissue dissolution^25^ and, consequently, disinfection after instrumentation. Its benefits rely on the transmission of acoustic energy from a “noncutting” and oscillating tip to an irrigant inside the root canal. The energy transmitted might lead to an acoustic streaming, cavitation, and/or warming of the irrigating substance, expanding its spectrum of action, especially on microorganisms in difficult-to-reach areas.^26^^,^^27^

This study showed the presence of cultivable bacteria in all initial samples (S1). Data showed the use of both protocols reduced the number of cultivable bacteria after single-file reciprocation technique, using 2.5% NaOCl as irrigant (S2), in both final irrigation protocols. However, the number of cultivable bacteria significantly decreased in PUA group, with 98.37% reduction percentage when comparing with CNI group, which only reduced 23.56%. Therefore, cultivable bacteria significantly reduced when comparing the protocols. After S3, PUA and CNI group resulted in 80% and 30% root canals free of cultivable bacteria, respectively. These results corroborate the findings in previous studies,^28^^,^^29^ which also observed a higher antibacterial effect using the irrigation solution associated with ultrasonic activation due to the cavitation promoted by the action of ultrasound on the irrigant.^13^

Although the literature shows PUA activation time may range from 20 seconds to 5 minutes,^28^^–^^30^ it has also demonstrated that a 30-second ultrasonic activation seems to be sufficient to achieve cleaner canals.^31^^,^^32^ The protocol established in this study was 2 cycles of 30 seconds of ultrasonic activation with 2.5% NaOCl while 17% EDTA was activated for 1 minute straight, resulting in a total of 2 minutes of ultrasonic activation. According to Van der Sluis, et al.^33^ (2009), the refreshment of the irrigant substance aids on dental debridement. Besides, emphasizing the importance of using both substances to remove smear layer is relevant, once it is known that neither NaOCl nor EDTA can alone eliminate both organic and inorganic portion of the smear layer.^34^

Some authors have drawn attention to the polymicrobial nature of endodontic infections.^9^^,^^35^^,^^36^ Supporting this statement, our study showed a mean of 9.6 species *per* root canal in the baseline (S1) using the checkerboard DNA-DNA hybridization method. *S. constellatus* was the most prevalent species before endodontic treatment, detected in 50% of all initial samples, followed by *E. faecalis* (45%), *F. nucleatum SP* (45%), *P. gingivalis* (45%), *P. melaninogenica* (45%), and *S. intermedius* (45%).

*S. constellatus, S. intermedius,* and *E. faecalis* remained in more than 45% of root canals in both groups, PUA e CNI, after endodontic treatment (S3). Likewise, these findings demonstrate that Gram-positive bacteria might be more resistant to endodontic treatment, as in Rôças and Siqueira^35^ (2011). Besides, *S. constellatus* and *S. intermedius* are highly prevalent in primary endodontic infections, and, despite being commensal oral bacteria, they may be related to acute and invasive diseases when associated.^37^ *E. faecalis* is also highly prevalent in primary endodontic infections due to its capacity to deeply penetrate into dentinal tubules^38^ and its resistance to intracanal medication, thus being considered a microorganism highly resistant to endodontic treatment. Although enterococci are not considered highly virulent microorganisms, some authors suggest their pathogenicity can be more related to its resistance to several antimicrobial agents.^39^^,^^40^ Moreover, synergistic interactions must be considered since their collective pathogenicity probably resulted from a combination of virulence factors.^36^ The authors understand the similarity between groups, considering the checkerboard results (bacterial species identification), and therefore, a supposed similarity of our results to the CFU data may be questioned. This disparity between our outcomes (CFU × checkerboard) might be explained by two reasons: 1- the outcome is different due to the specificity of the analysis, or 2- due to the sample size used for checkerboard analysis. As the checkerboard was a complementary analysis in this study, one might assume it did not influence negatively the study. On the other hand, checkerboard, when used to detect microbiological profile between different types of endodontic infection, must be used as the main outcome and included in the sample size.

In this study, the primary outcome measurement was defined by CBCT analysis, which suggested both groups were effective in reducing periapical lesion. Moreover, both treatments resulted in clinical success considering the absence of pain, mobility, and fistula. As the power estimation to include patients considered the volume assessment, the above clinical considerations might be underpowered for granting such comparison, despite being an important outcome for the proposed treatments.

## Conclusion

In conclusion, both treatments significantly decreased the number of bacterial species when compared with the initial sample. However, no statistical difference in the total microbial load between PUA and CNI groups was detected. The number of cultivable anaerobic bacteria significantly decreased using PUA; bacterial composition and number of bacterial species found after using CNI or PUA was similar.
